# Single-cell transcriptome analysis of NEUROG3+ cells during pancreatic endocrine differentiation with small molecules

**DOI:** 10.1186/s13287-023-03338-z

**Published:** 2023-04-25

**Authors:** Jin Li, Junru Chen, Xiaoyu Luo, Guangxiu Lu, Ge Lin

**Affiliations:** 1 Institute of Reproductive and Stem Cell Engineering, School of Basic Medical Science, Central South University, Changsha, 410078 Hunan People’s Republic of China; 2 Key Laboratory of Stem Cells and Reproductive Engineering, Ministry of Health, Changsha, 410078 Hunan People’s Republic of China; 3 National Engineering and Research Center of Human Stem Cells, Changsha, 410078 Hunan People’s Republic of China; 4 Reproductive and Genetic Hospital of CITIC-Xiangya, Changsha, 410008 Hunan People’s Republic of China

**Keywords:** Pancreatic endocrine cells, hESCs, DAPT + 4FS, NEUROG3

## Abstract

The efficiency of inducing human embryonic stem cells into NEUROG3+ pancreatic endocrine cells is a bottleneck in stem cell therapy for diabetes. To understand the cell properties and fate decisions during differentiation, we analyzed the modified induction method using single-cell transcriptome and found that DAPT combined with four factors (4FS): nicotinamide, dexamethasone, forskolin and Alk5 inhibitor II (DAPT + 4FS) increased the expression of NEUROG3 to approximately 34.3%. The increased NEUROG3+ cells were mainly concentrated in Insulin + Glucagon + (INS + GCG+) and SLAC18A1 + Chromogranin A+(SLAC18A1 + CHGA +) populations, indicating that the increased NEUROG3+ cells promoted the differentiation of pancreatic endocrine cells and enterochromaffin-like cells. Single-cell transcriptome analysis provided valuable clues for further screening of pancreatic endocrine cells and differentiation of pancreatic islet cells. The gene set enrichment analysis (GSEA) suggest that we can try to promote the expression of INS + GCG+ population by up-regulating G protein-coupled receptor (GPCR) and mitogen-activated protein kinase signals and down-regulating Wnt, NIK/NF-KappaB and cytokine-mediated signal pathways. We can also try to regulate GPCR signaling through PLCE1, so as to increase the proportion of NEUROG3+ cells in INS+GCG+ populations. To exclude non-pancreatic endocrine cells, ALCAM^high^ CD9^low^ could be used as a marker for endocrine populations, and ALCAM^high^ CD9^low^CDH1^low^ could remove the SLC18A1 + CHGA+ population.

**Supplementary Information:**

The online version contains supplementary material available at 10.1186/s13287-023-03338-z.

## Letter

**Fig. 1:**
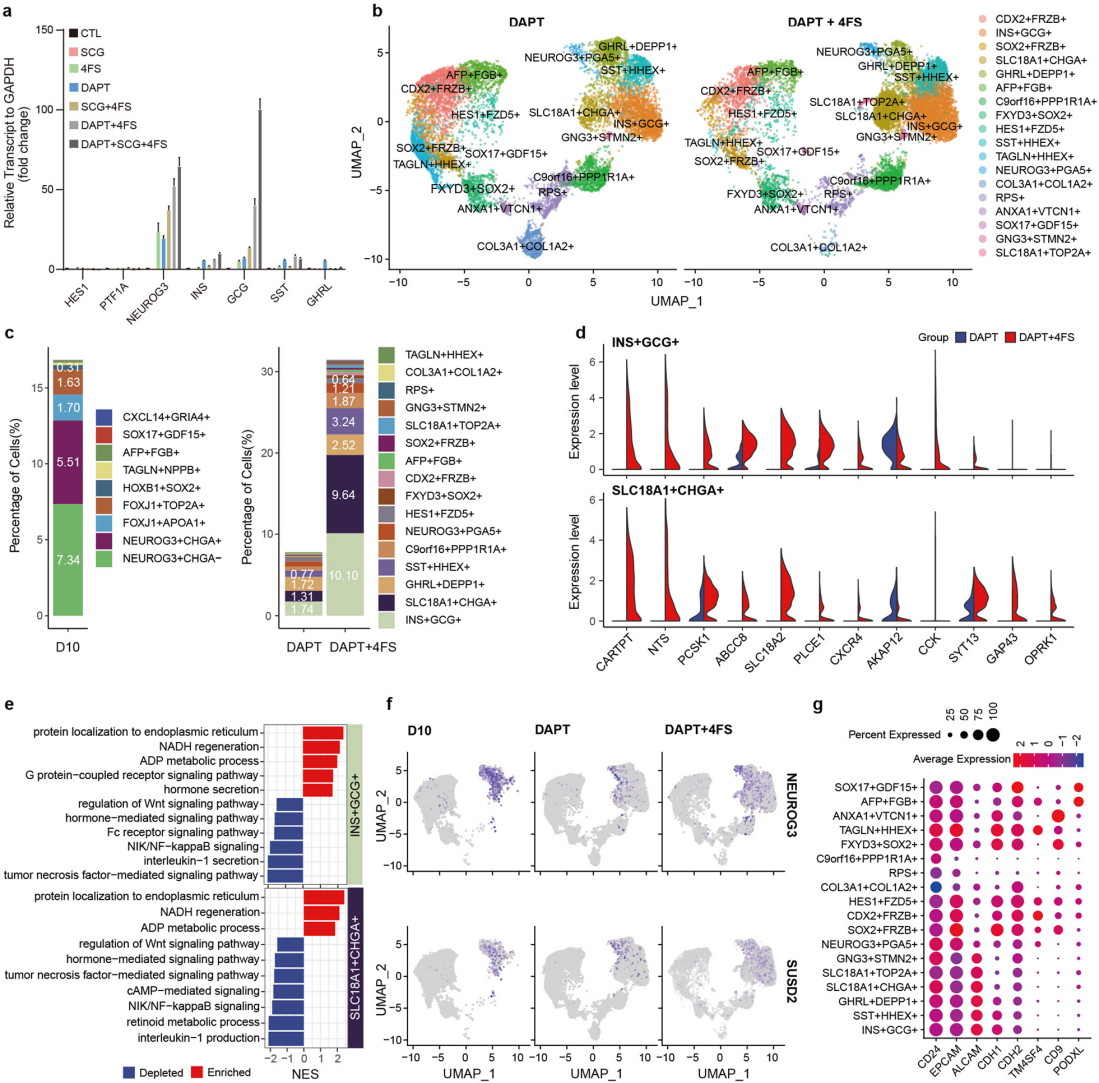
Pancreatic endocrine cell differentiation under conditions of DAPT with DAPT + 4FS. **a** NEUROG3 and islet markers were promoted by various small molecules. DAPT is a γ-secretase inhibitor and inhibits NOTCH signaling in vitro. SCG: sodium cromoglycate. 4FS indicates a combination of nicotinamide, dexamethasone, forskolin, and Alk5 inhibitor II. The primers for QPCR are shown in Additional file 7: Table S1. **b** Uniform Manifold Approximation and Projection (UAMP) shows populations between DAPT and DAPT + 4FS on day 14. **c** Proportion and distribution of NEUROG3+ cells on day 10 and day 14. **d** Violin plot shows the various genes among NEUROG3 + cells in INS + GCG+ and SLC18A1 + CHGA+ populations. Enriched and depleted refer to the comparative results of DAPT + 4FS compared to DAPT. **e** GSEA results of NEUROG3+ cells in INS + GCG+ and SLC18A1 + CHGA+ populations. Enriched and depleted refer to the comparison results of DAPT + 4FS compared to DAPT. **f** Feature plot shows the expression and distribution of NEUROG3 and SUSD2. **g** Dot plot shows surface markers in DAPT + 4FS, ALCAM^high^ CDH1^high^CD9^low^ that can be used as markers for the pancreatic endocrine population

Inducing hESCs into islet-like cells proceeds through four stages, including definitive endoderm, pancreatic precursor, pancreatic endocrine, and islets. However, the induction efficiency of NEUROG3+ pancreatic endocrine cells was low. Liu et al. [1] reported that the percentage of NEUROG3+ cells was only 12%. Kondo et al. [2] proved that sodium cromoglicate (SCG) combined with 4FS (SCG+4FS) increased the proportion of NEUROG3+ cells to 32%. DAPT had been proved to inhibit the Notch signal pathway and promote the endocrine differentiation of pancreas [3]. In the current study, we verified the effects of DAPT, SCG and 4FS (Additional file 1) and found that SCG alone had little effect, while DAPT alone could effectively promote the expression of NEUROG3, INS, GXG, Somatostatin(SST) and Ghrelin(GHRL). At the same time, we found that DAPT + 4FS had a stronger ability to promote the expression of NEUROG3, INS, GXG and SST than SCG + 4FS group (Fig. 1a). These results suggested that both DAPT group and DAPT + 4FS group could promote the expression of NEUROG3, INS, GXG and SST, and DAPT + 4FS group could increase NEUROG3+ cells from 15.7% to about 34.3% compared with DAPT group (Additional file 2: Fig. S1).

Single-cell transcriptome analysis allows to follow NEUROG3+ cells during differentiation. NEUROG3+ cells on day 10 were divided into two classes: NEUROG3+CHGA− population expressed PRSS1, TTR, APOA2 and IGFBP2; NEUROG3+CHGA+ population expressed CHGA, PEG10, SCG2 and SCGN (Additional file 3: Fig. S2). NEUROG3+CHGA+ cells initiate the differentiation of endocrine cells. The increase of NEUROG3+ cells on day 14 in DAPT + 4FS compared to DAPT was mainly concentrated in INS + GCG+ (from 1.74 to 10.10%) and SLAC18A1 + CHGA+ (from 1.31 to 9.64%) populations (Fig. 1b and c). This increase trend was consistent with our previous results by flow cytometry. The SLAC18A1 + CHGA+ population expressed markers of enterochromaffin-like cells [4] such as SLC18A1, FEV and COL8A1 (Additional file 4: Fig. S3a and d). These results indicated that the increase of NEUROG3+ cells was not limited to pancreatic endocrine cells but also promoted the differentiation of enterochromaffin-like cells.

Among NEUROG3+ cells in INS + GCG+ and SLAC18A1 + CHGA+ populations, secretory function-related markers such as ABCC8, SLC18A2 and PCSK1 were significantly upregulated in both populations under DAPT + 4FS, while PLCE1, CXCR4, AKAP12 and CCK were upregulated under DAPT + 4FS compared to DAPT in INS + GCG+ population. SYT13, GAP43 and OPRK1 were also upregulated under DAPT + 4FS compared to DAPT in SLC18A1 + CHGA+ population (Fig. 1d). We attempted to analyze the different signals of NEUROG3+ cells in these two populations to understand the possible reasons for the increase of NEUROG3+ cells. The GSEA results showed that the signals of NADH regeneration and ADP metabolism process were increased, and the Wnt, NF-kB signals were decreased in both populations. In addition, GPCR signal was upregulated in INS + GCG+ population (Fig. 1e), suggesting that the upregulation of GPCR signals might be related to the increase of NEUROG3+ cells in INS + GCG+ population. Previous analysis showed that PLCE1 was significantly upregulated in INS + GCG+ population (Fig. 1d), which is related to GPCR signaling and involved in islet Ca^2+^ regulation and insulin release [5]. It was suggested that PLCE1 might play an important role in promoting the increase of NEUROG3+ cells in INS + GCG+ population by regulating GPCR signaling.

We found that three islet endocrine populations on day14 accounted for 30.8% under DAPT and 40.35% under DAPT + 4FS, suggesting that DAPT + 4FS promoted pancreatic endocrine differentiation. Three islet endocrine populations expressed islet β-cells marker INS, α-cells marker GCG, δ-cells marker SST, and ε-cells marker GHRL but not the PP-cell marker pancreatic polypeptide (PPY) (Additional file 4: Fig. S3a and c). Moreover, INS + GCG + and SST + HHEX+ populations under DAPT + 4FS increased by 9.36% and 3.65%, whereas GHRL + DEPP1+ population under DAPT + 4FS decreased by 3.47% (Additional file 4: Figure S3b). These results suggested that DAPT + 4FS promoted the differentiation of β-cells, α-cells and δ-cells and decreased ε-cells. To identify the possible clues for the regulation of differentiation of INS + GCG+ and SLC18A1 + CHGA+ populations under DAPT + 4FS, we first compared the signals between these two populations. The GSEA results showed that the INS + GCG+ population upregulated GPCR and MAPK signals (Additional file 4: Fig. S3e). Next, we compared the signals between INS + GCG+ population and NEUROG3+ cells on day 10, GSEA results also showed that GPCR and MAPK signals were up-regulated and Wnt, NF-kB and cytokine-mediated signal pathways were down-regulated in INS + GCG+ population (Additional file 5: Fig. S4). It was suggested that the up-regulation of GPCR and MAPK signals and down-regulation of Wnt, NF-kB and cytokine-mediated signal pathways might be related to the increase of INS + GCG+ population.

However, the SLC18A1 + CHGA+ population under DAPT + 4FS increased from 4 to 16.25% compared with DAPT, and the GSEA results between SLC18A1 + CHGA+ population and NEUROG3+ population on day10 showed that GPCR and MAPK signals were up-regulated in SLC18A1 + CHGA+ population, while Wnt and NF-kB were down-regulated (Additional file 5: Fig. S4). This change of signal regulation pathway in SLC18A1 + CHGA+ population showed a similar way to that in INS + GCG+ population, but the level of up-regulation of GPCR and MAPK signaling in INS + GCG+ population was stronger than that in SLC18A1 + CHGA+ population. Therefore, the removal of enterochromaffin-like cells (SLC18A1 + CHGA+) was important for islet differentiation. SLC18A1+ cells were first appeared in NEUROG3 + CHGA+ population (Additional file 4: Fig. S3c), following which we sought to identify surface markers for the SLC18A1 + CHGA+ population and analyzed the pancreatic endocrine surface markers such as CD200 [6], SUSD2 [1], and PROM1 [7] (Additional file 6: Fig. S5a, b). Although the distribution of SUSD2+ cells was consistent with that of NEUROG3+ cells (Fig. 1f and Additional file 6: Fig. S5a), they were not capable of removing the SLC18A1 + CHGA+ population. Strikingly, we found that ALCAM^high^ CD9^low^ [8] can be used as a marker for endocrine populations, and using ALCAM^high^ CD9^low^CDH1^low^ could remove the SLC18A1 + CHGA+ population (Fig. 1g and Additional file 6: Fig. S5c).

Single-cell transcriptome analysis provides some valuable clues for improving the induction protocol. We can try to promote the expression of INS + GCG+ population by up-regulating GPCR and MAPK signals and down-regulating the signal pathway mediated by Wnt, NF-kB and cytokines. We can also try to regulate GPCR signals through PLCE1 to promote NEUROG3+ cells in INS + GCG+ population. For non-islet endocrine cells, we can try to use ALCAM^high^ CD9^low^CDH1^low^ to remove SLC18A1 + CHGA+ population.

## Supplementary Information

**Additional file 1.** Methods used in this study.

**Additional file 2. Fig. S1**: Expression of pancreatic and duodenal homeobox 1and NEUROG3 under DAPT VS DAPT+4FS on day 14. a Expression of PDX1and NEUROG3by immunofluorescence. b Flow cytometry showed that 16.4 ± 0.6% NEUROG3+ cells in DAPT, while DAPT+4FS could increase NEUROG3+ cells to 35.3 ± 5.0%.

**Additional file 3. Fig. S2**: Cell characteristics on day10. a Single-cell RNA sequencing analysis of the sample confirmed nine populations and revealed two classes of NEUROG3+ cells: NEUROG3+ CHGA+ and NEUROG3+CHGA- populations. b Dot plot shows the markers of the different populations. c The proportion of populations on day 10 and two main populations FOXJ1+APOA1+ and FOXJ1+TOP2A+ accounted for 47.97% and 19.30%, respectively. In addition, there were 3.48% mesoderm cellsand 7.17% ectoderm cells. d Gene expression of NEUROG3+ cells between NEUROG3+CHGA+ and NEUROG3+CHGA- populations.

**Additional file 4. Fig. S3**: Cell characteristics on day14. a Dot plot shows markers of eighteen populations. b The proportion of populations on day 14. With the determination of SOX2/CDX2 axis and cells was divided into anterior foregut, midgut/hindgutand pancreas exocrine. The populations of midgut/hindgutand liver cellsin DAPT+4FS decreased by 5.84% and 2.87%. In addition, DAPT+4FS decreased muscle cellsfrom 7.31 to 0.37% and mesenchymal cellsfrom 6.58 to 0.44%, suggesting that DAPT+4FS inhibited the differentiation of mesodermal cells. c Feature plot shows the distribution of INS, GCG, SST, GHRL, PPY and SLC18A1 on day 14. d Marker genes of INS+GCG+ and SLAC18A1+CHGA+ populations under DAPT+4FS, up and down refer to the comparison result of INS+GCG+ population relative to SLAC18A1+CHGA+ population. e GSEA results between INS+GCG+ and SLC18A1+CHGA+ populations.

**Additional file 5. Fig. S4**: GSEA results between populations under DAPT+4FS on day 14 and NEUROG3+ populations on day 10. Enriched and depleted refer to the comparative results of five populations in DAPT+4FScompared to two NEUROG3+ populationson day 10.

**Additional file 6. Fig. S5**: Surface markers for NEUROG3+ cells and pancreatic endocrine cells. a The percentage of NEUROG3+ and SUSD2+ cells on day 10 and day14 under DAPT+4FS, respectively. b Feature plot shows the distribution of CD200 and PROM1. c Feature plot shows distribution of surface markers related to pancreatic endocrine cells.

**Additional file 7. Table S1**. The primers for Q-PCR.

## Data Availability

The data set supporting the results of this article has been deposited in the EMBL European Nucleotide Archive (ENA) under BioProject accession code PRJEB55509.

## Abbreviations

hESCs: Human embryonic stem cells
SCG: Sodium cromoglycate
4FS: 4 Factors
GSEA: Gene set enrichment analysis
UAMP: Uniform Manifold Approximation and Projection
GPCR: G protein-coupled receptor
MAPK: Mitogen-activated protein kinase
NF-kB: NIK/NF-KappaB
PDX1: Pancreatic and duodenal homeobox 1
