# Supplementary material for: Single-cell transcriptome analysis of NEUROG3+ cells during pancreatic endocrine differentiation with small molecules
Source: Stem Cell Res Ther. 2023 Apr 25;14:101. doi: 10.1186/s13287-023-03338-z (PMC10127065; doi:10.1186/s13287-023-03338-z)
Supplement: Supplementary file 1 — Additional file 1. Methods used in this study. [file 13287_2023_3338_MOESM1_ESM.docx]

Methods

Cell culture and differentiation

HESCs were selected from the stem cell bank of the National Engineering and Research Center of Human Stem Cells. In preparation for differentiation, hESCs were cultured in 6-wells plates coated with Matrigel (BD, 354277) and containing mTeSR (Stem Cell, 085850) medium for 3-4 days. Until the confluence of the cells reached more than 80%, we initiated differentiation. The differentiation scheme were devided into three stages. In stage 1 for the definitive endoderm, 100 ng/ml Activin A (R&D, 338-ac-050) and 3 μM CHIR99021 (Stemgent, 04-0004) were added to RPMI1640 (Thermo Fisher, 11875) medium containing 2% fetal bovine serum (FBS, Invitrogen) for 24 h and then to RPMI 1640 medium containing 2% FBS and 100 ng/ml Activin A for 48 h. In stage 2 for pancreatic progenitor cells differentiation, 2μM retinoic acid (Stemgent, 04-0021), 10μM SB431542 (Stemgent, 04-0010-10) and 1μM Dorsomorphin (Stemgent, 04-0024) were added in IMEM (Thermo Fisher, A1048901) medium containing 1% B27 (Invitrogen) for 7 days. In stage 3, there were two different options for pancreatic endocrine differentiation from day 10 to day 14, referred to as DAPT and DAPT+4FS. DAPT means that cells were treated with 1μM DAPT (Stemgent, 04-0041) alone for 4 days; DAPT+4FS means that 1μM DAPT was synergized with 10 mM nicotinamide (Sigma, N0636-500G), 10 μM dexamethasone (Wako,047-18863), 10μM forskolin (Wako,063-02193) and 5 μM Alk5 inhibitor II (Biogems,4463325-10) for 4 days. Both options use IMEM (Thermo Fisher, A1048901) medium with 1% B27 (Invitrogen).

Immunofluorescence staining

Cells were collected on day 14 for immunofluorescence staining. Samples were fixed with 4% paraformaldehyde for 15 minutes at room temperature and then incubated with 5% Donkey Serum (Jackson ImmunoResearch, 017-000-121) for 30 minutes at room temperature. The primary antibody was incubated overnight at 4 ℃, and the secondary antibody was incubated on the next day at room temperature for 2 hours. Samples were then stained with DAPI for 3-5 minutes and placed under a fluorescence microscope for observation. The primary antibody information as follows: PDX1 (Abcam, ab47308), NEUROG3 (R&D, AF3444). The secondary antibody was obtained from Jackson ImmunoResearch and Invitrogen, respectively.

Flow cytometry

Cells collected on day 14 were fixed with a fixation / permeabilization solution (BD, 554714) for 15 minutes and then stained with an antibody. The intermediate- labeled antibody NEUROG3 (R&D, AF3444) was incubated overnight at 4 ℃, and the secondary antibody was incubated on the next day at room temperature for 1 hours. The cell suspension was passed through a 100-um filter and resuspended in FACS solution for subsequent flow acquisition.

Real-time fluorescence quantitative PCR(Q-PCR)

Cells were collected and lysed by adding 1 ml of Trizol (Life, 15596026), and then cDNA template was obtained using reverse transcription Kit (Roche, 04896966001). Q-PCR samples were detected with 480 fluorescent dyes (Roche, 4887352001-1).

10X single cell transcriptome sequencing analysis

Samples were collected on day 10 (named day 10)and day 14 (named DAPT and DAPT+4FS) and delivered to Shanghai Jing Neng Company for 10X single-cell sequencing analysis. Cell suspension was loaded into Chromium microfluidic chips with 3’v3 chemistry and barcoded with a 10X Chromium Controller (10X Genomics). RNA from the barcoded cells was then reverse-transcribed and sequencing libraries constructed with reagents from a Chromium Single Cell 3’ v3 reagent kit (10X Genomics) according to the manufacturer’s instructions. Sequencing was performed using Illumina NovaSeq 6000 (Illumina).

Single-cell RNA analysis

The cell range data provided by Shanghai Jing Neng Company was analyzed by the bioinformatics analysis team of Reproductive & Genetic Hospital of CITIC Xiang Ya. Raw reads were demultiplexed and mapped to the human reference genome with the Cell Ranger (10X Genomics) pipeline (version 4.0.0) using default parameters. The generated gene-cell expression matrix was used for subsequent analysis in R (version 4.0.2) using Seurat (version 4.0.2). “Cells” fit any of the following criteria were excluded: < 500 expressed genes, > 6000 expressed genes, < 300 UMIs (unique molecular identifiers), > 5% mitochondrial gene percentage. The filtered cells were used for downstream graph-based clustering adapting the typical pipeline. Briefly, gene expression was normalized using the “NormalizeData” function. Highly variable genes were calculated using the “FindVariableGenes” method (parameters: selection.method = “vst”, nfeatures = 2000). The variables "percent.mt", "S.Score" and "G2M.Score" were regressed out in the scaling step. The top 20 principal components were used for downstream graph-based clustering into different populations (FindClusters function), and UMAP visualization. Different expression genes (DEGs) in each cluster were identified with the function “FindMarkers” in Seurat (parameters: min.pct = 0.1, logfc. threshold=0) and the cutoff criteria (|avg_log2FC|>0.25 and P value<0.05). Gene set enrichment analysis (GSEA) was performed using the clusterProfiler package (version 3.16.1) in R based on GO database and all genes (min.pct = 0.1). The “DoHeatmap” function in Seurat was used to obtain heatmap mappings. The “FeaturePlot” and “DotPlot” functions in Seurat were used for the visualize the expression of specific genes. Other plots were visualized using package gghalves (version 0.1.1) and ggplot2 (version 3.3.5) in R.
