# Supplementary figures and images for: Single-cell transcriptome analysis of NEUROG3+ cells during pancreatic endocrine differentiation with small molecules
Source: Stem Cell Res Ther. 2023 Apr 25;14:101. doi: 10.1186/s13287-023-03338-z (PMC10127065; doi:10.1186/s13287-023-03338-z)

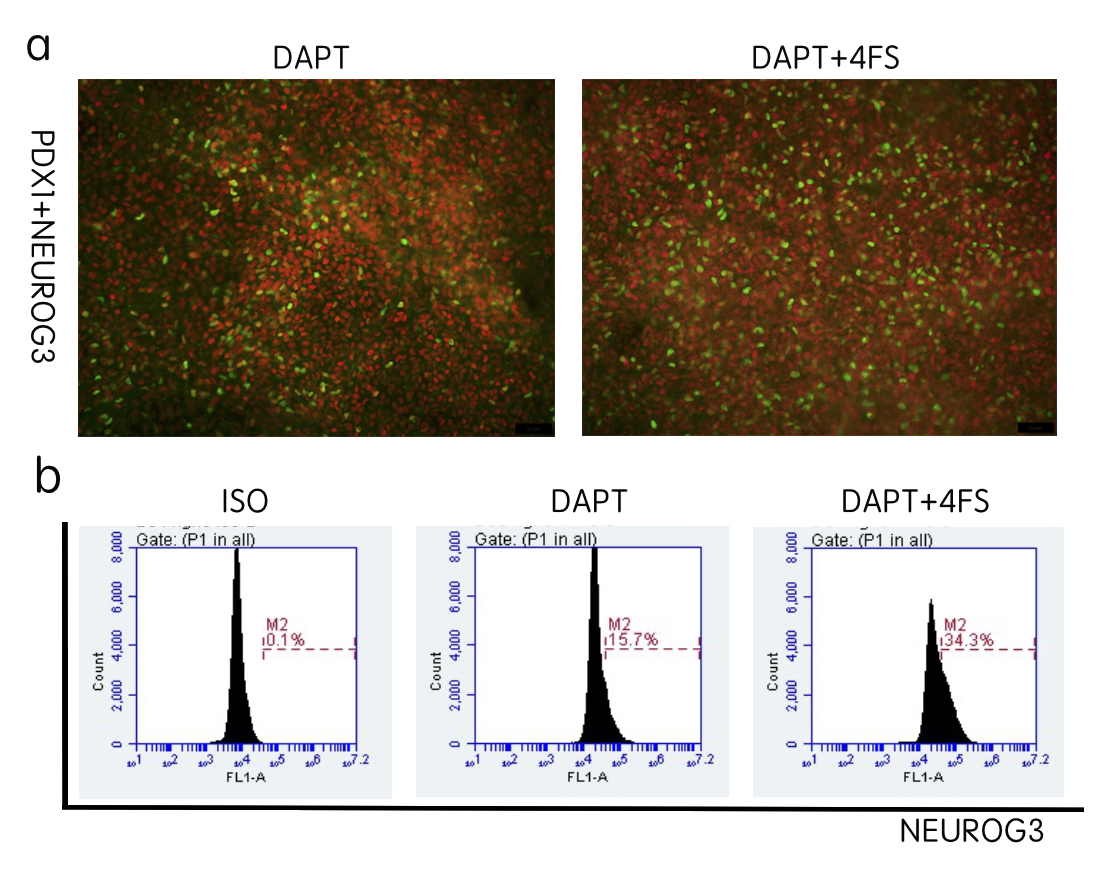

Supplement: Supplementary file 2 — Additional file 2. Fig. S1: Expression of pancreatic and duodenal homeobox 1and NEUROG3 under DAPT VS DAPT+4FS on day 14. a Expression of PDX1and NEUROG3by immunofluorescence. b Flow cytometry showed that 16.4 ± 0.6% NEUROG3+ cells in DAPT, while DAPT+4FS could increase NEUROG3+ cells to 35.3 ± 5.0%. [file 13287_2023_3338_MOESM2_ESM.png]

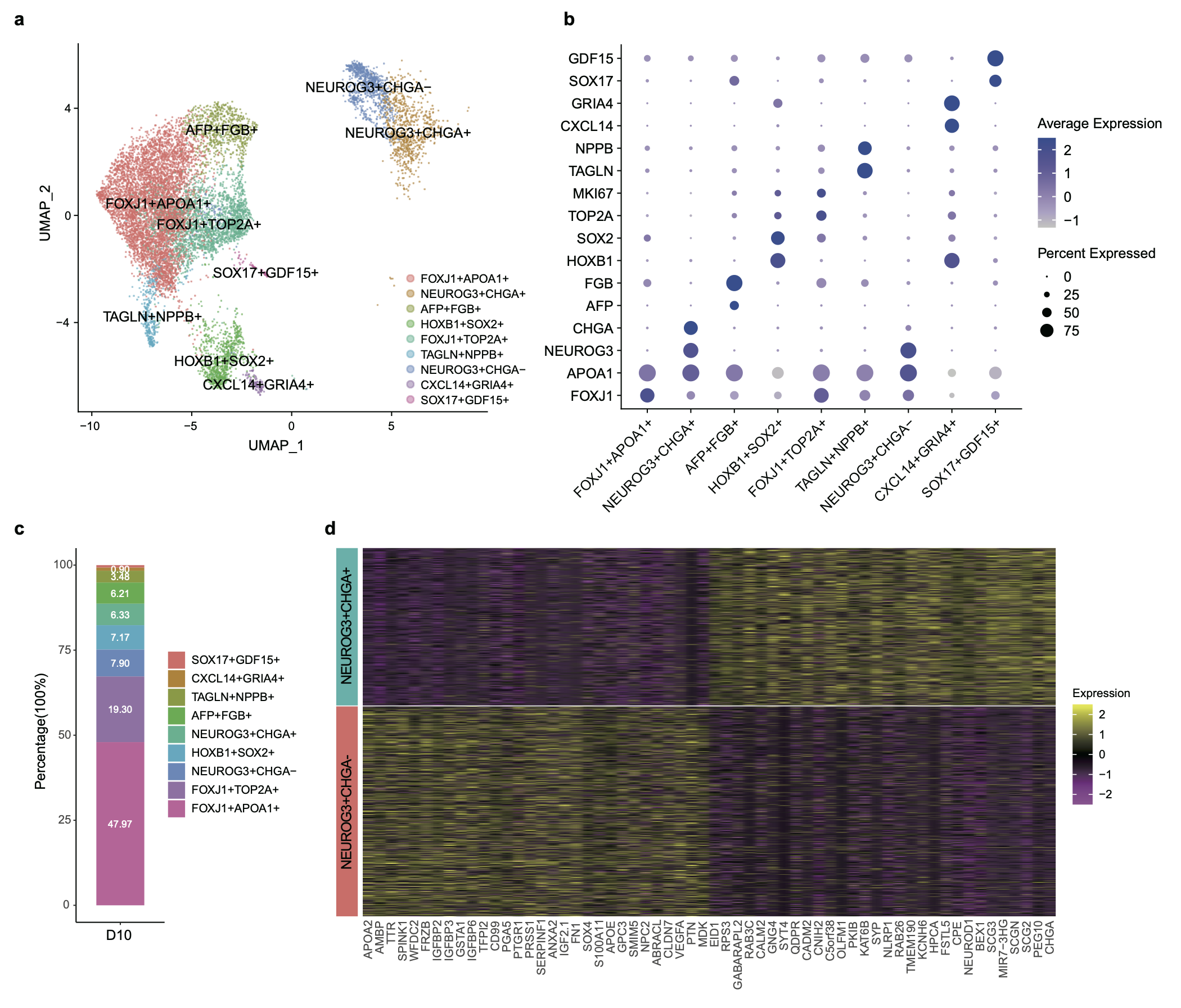

Supplement: Supplementary file 3 — Additional file 3. Fig. S2: Cell characteristics on day10. a Single-cell RNA sequencing analysis of the sample confirmed nine populations and revealed two classes of NEUROG3+ cells: NEUROG3+ CHGA+ and NEUROG3+CHGA- populations. b Dot plot shows the markers of the different populations. c The proportion of populations on day 10 and two main populations FOXJ1+APOA1+ and FOXJ1+TOP2A+ accounted for 47.97% and 19.30%, respectively. In addition, there were 3.48% mesoderm cellsand 7.17% ectoderm cells. d Gene expression of NEUROG3+ cells between NEUROG3+CHGA+ and NEUROG3+CHGA- populations. [file 13287_2023_3338_MOESM3_ESM.png]

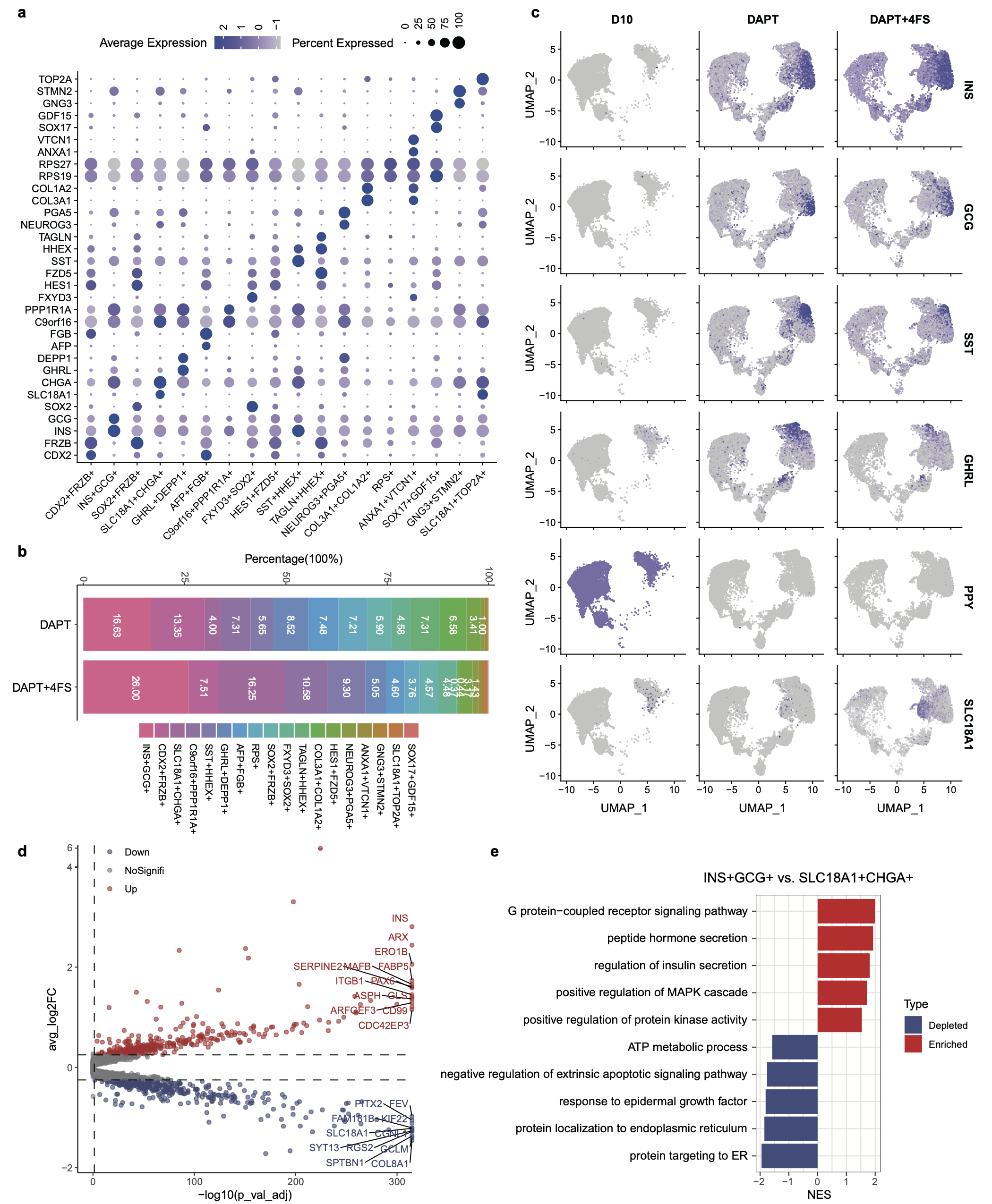

Supplement: Supplementary file 4 — Additional file 4. Fig. S3: Cell characteristics on day14. a Dot plot shows markers of eighteen populations. b The proportion of populations on day 14. With the determination of SOX2/CDX2 axis and cells was divided into anterior foregut, midgut/hindgutand pancreas exocrine. The populations of midgut/hindgutand liver cellsin DAPT+4FS decreased by 5.84% and 2.87%. In addition, DAPT+4FS decreased muscle cellsfrom 7.31 to 0.37% and mesenchymal cellsfrom 6.58 to 0.44%, suggesting that DAPT+4FS inhibited the differentiation of mesodermal cells. c Feature plot shows the distribution of INS, GCG, SST, GHRL, PPY and SLC18A1 on day 14. d Marker genes of INS+GCG+ and SLAC18A1+CHGA+ populations under DAPT+4FS, up and down refer to the comparison result of INS+GCG+ population relative to SLAC18A1+CHGA+ population. e GSEA results between INS+GCG+ and SLC18A1+CHGA+ populations. [file 13287_2023_3338_MOESM4_ESM.png]

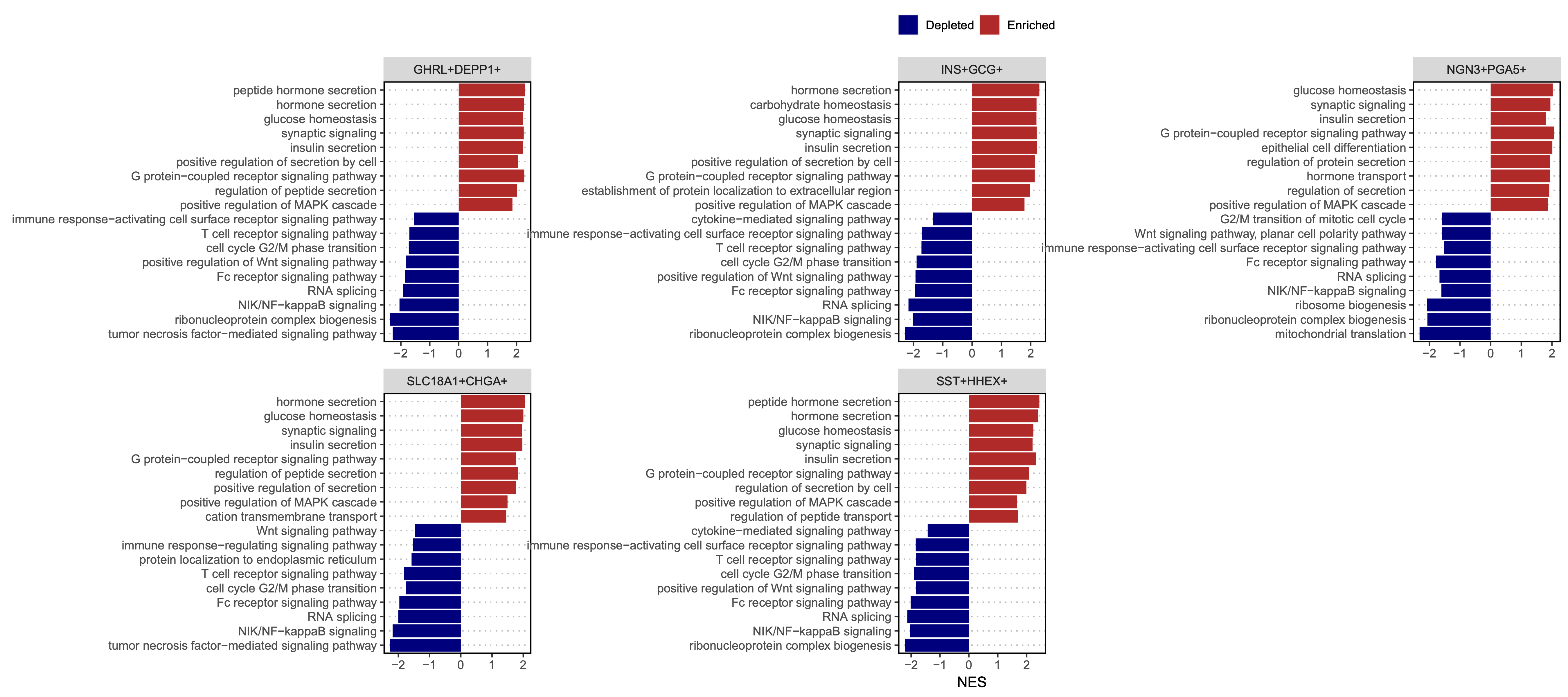

Supplement: Supplementary file 5 — Additional file 5. Fig. S4: GSEA results between populations under DAPT+4FS on day 14 and NEUROG3+ populations on day 10. Enriched and depleted refer to the comparative results of five populations in DAPT+4FScompared to two NEUROG3+ populationson day 10. [file 13287_2023_3338_MOESM5_ESM.png]

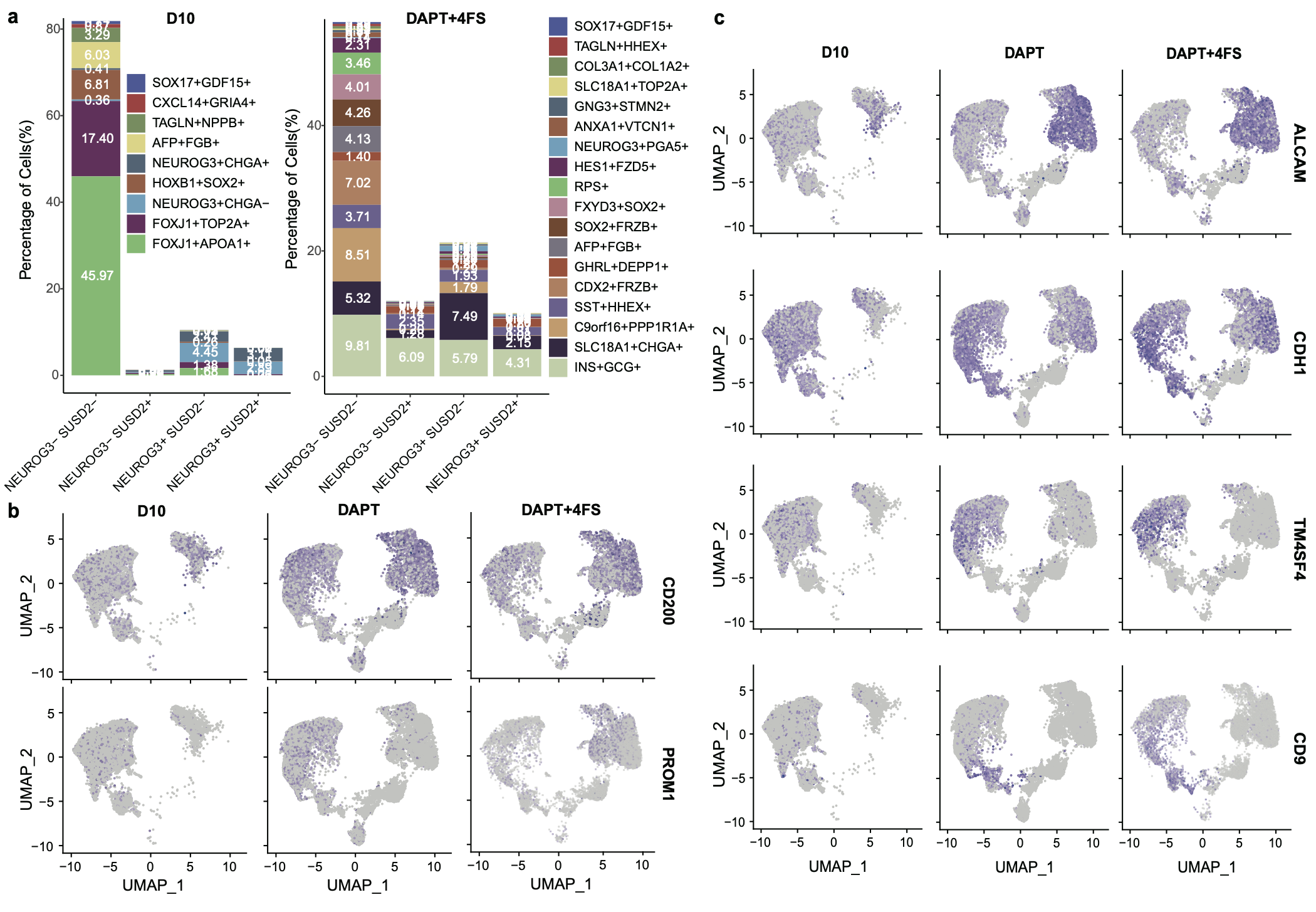

Supplement: Supplementary file 6 — Additional file 6. Fig. S5: Surface markers for NEUROG3+ cells and pancreatic endocrine cells. a The percentage of NEUROG3+ and SUSD2+ cells on day 10 and day14 under DAPT+4FS, respectively. b Feature plot shows the distribution of CD200 and PROM1. c Feature plot shows distribution of surface markers related to pancreatic endocrine cells. [file 13287_2023_3338_MOESM6_ESM.png]
