# Supplementary material for: Single-cell transcriptome analysis of NEUROG3+ cells during pancreatic endocrine differentiation with small molecules
Source: Stem Cell Res Ther. 2023 Apr 25;14:101. doi: 10.1186/s13287-023-03338-z (PMC10127065; doi:10.1186/s13287-023-03338-z)
Supplement: Supplementary file 7 — Additional file 7. Table S1. The primers for Q-PCR. [file 13287_2023_3338_MOESM7_ESM.docx]

Supplementary Table S1. The primers for QPCR

| Gene Name | Primers (Forward/Reverse; 5’-to-3’) |
| --- | --- |
| HES1-F | GGCGGACATTCTGGAAATGA |
| HES1-R | GTCACCTCGTTCATGCACTC |
| PTF1A-F | GAAGGTCATCATCTGCCATCG |
| PTF1A-R | GGCCATAATCAGGGTCGCT |
| NEUROG3-f | GCTCATCGCTCTCTATTCTTTTGC |
| NEUROG3-R | GGTTGAGGCGTCATCCTTTCT |
| INS-F | GGGGAACGAGGCTTCTTCTA |
| INS-R | TGTTCCACAATGCCACGC |
| SST-F | CGCTGTCCATCGTCCTG |
| SST-R | GGGCATCATTCTCCGTCTG |
| GCG-F | GAGACATGCTGAAGGGACCT |
| GCG-R | TCAACAATGGCGACCTCTTC |
| GHRL-F | ATGGAGGTCAAGCAGAAGGG |
| GHRL-R | CTGAAGAAACTTCCCCAGGG |
| GAPDH-F | GTCAAGGCTGAGAACGGGAA |
| GAPDH-R | TCGCCCCACTTGATTTTGGA |
